# Mediating role of cognition and social cognition on creativity among patients with schizophrenia and healthy controls: Revisiting the Shared Vulnerability Model

**DOI:** 10.1111/pcn.12954

**Published:** 2019-12-04

**Authors:** Agurne Sampedro, Javier Peña, Naroa Ibarretxe‐Bilbao, Pedro Sánchez, Nagore Iriarte‐Yoller, Sara Ledesma‐González, Mikel Tous‐Espelosin, Natalia Ojeda

**Affiliations:** ^1^ Department of Methods and Experimental Psychology, Faculty of Psychology and Education University of Deusto Bilbao Spain; ^2^ Refractory Psychosis Unit, Hospital Psiquiátrico de Alava Vitoria Spain; ^3^ Department of Neuroscience, Psychiatry Section, School of Medicine and Odontology University of the Basque Country (UPV/EHU) Vizcaya Spain; ^4^ Department of Physical Education and Sport, Faculty of Education and Sport University of the Basque Country (UPV/EHU) Vitoria‐Gasteiz Spain

**Keywords:** cognition, creativity, executive function, schizophrenia, theory of mind

## Abstract

**Aim:**

As suggested by the Shared Vulnerability Model, impairment in executive functions could lead to worse creative performance among individuals with schizophrenia. Another impaired function in schizophrenia, previously related to creativity in healthy people, is theory of mind. However, little is known about the effect of theory of mind in creativity in schizophrenia. Therefore, the aim of this study was to analyze differences in creativity among patients with schizophrenia compared to healthy controls (HC) and to explore the potential role of executive functions and theory of mind as mediators of this relation.

**Methods:**

Forty‐five patients with schizophrenia and 45 HC underwent a neuropsychological assessment, including executive functions (cognitive flexibility and working memory), theory of mind, and verbal and figural creativity.

**Results:**

As expected, patients with schizophrenia obtained lower scores in creativity, cognitive flexibility, working memory, and theory of mind compared to HC. Path analysis showed that theory of mind mediated the relation between group (schizophrenia or HC) and both figural (*Z* = 2.075, *P* = 0.037) and verbal creativity (*Z* = 2.570, *P* = 0.010). Working memory mediated the relation between group and figural creativity (*Z* = 2.034, *P* = 0.041) and was marginally significant for verbal creativity (*Z* = 1.930, *P* = 0.053). Finally, cognitive flexibility mediated between group and figural creativity (*Z* = 2.454, *P* = 0.014).

**Conclusion:**

Results suggest that the lower performance in creativity among patients with schizophrenia was partly due to an impairment in executive functions and theory of mind. The involvement of theory of mind opens up a new field of research as a possible risk factor in the Shared Vulnerability Model.

The idea that creativity and psychopathology are related to each other dates back several centuries.^1^ This idea has been mainly reinforced by case studies,^2^, ^3^ such as those of famous geniuses who suffered from schizophrenic symptoms (e.g., Vincent Van Gogh and John Nash).^4^ However, empirical studies have obtained some contradictory findings. In fact, the majority of empirical studies available have reported worse creative performance among people with schizophrenia compared to healthy controls (HC),^4^, ^5^, ^6^ while very few studies have reported the opposite.^7^ In addition, in an epidemiological study carried out by Kyaga *et al*., people with schizophrenia did not show an increased rate of creative professions compared with controls.^8^ Furthermore, a recent meta‐analysis^9^ concluded that schizophrenia is negatively related to creativity. All this empirical evidence is consistent with the idea that creativity and psychopathology have an inverted U‐shape relation, which means that some minor schizotypal symptoms could promote creative thinking, but a greater severity of symptoms (such as symptoms present in schizophrenia) could impede it.^10^, ^11^ In the relation between creativity and psychopathology, Carson^10^ made an excellent contribution after proposing the Shared Vulnerability Model. According to this model, there are several genetic vulnerability factors common to both creativity and psychopathology that would promote accessibility to the associational material that is usually processed outside consciousness.^10^ This increased accessibility to associational material, combined with an adequate executive control and other cognitive functions, could lead to creativity.^10^ However, this enhanced accessibility, combined with an executive dysfunction, could instead constitute a risk of psychopathology. This model suggests that the risk factors that would promote psychopathology instead of creative thinking are low IQ, working memory (WM) deficits, and an altered cognitive flexibility (CF).^10^

The fact that multiple factors are involved in the relation between psychopathology and creativity may be partially due to the complexity of the concept of creativity itself. Creativity is usually defined as the ability to produce something original or novel and appropriate or useful for a task.^12^ One of the main components of creative thinking is divergent thinking, which is the ability to simultaneously activate and establish remote associations between unrelated concepts from distant categories, as well as to generate multiple alternative and novel answers to a problem.^13^ Divergent thinking is composed of several dimensions, including originality, fluency, flexibility, and elaboration,^13^ and it can be expressed in different modalities, such as in verbal or figural forms. Over the past few decades, different approaches concerning creative cognition, creative drives, and neuromodulatory circuits have been developed in order to understand the concept of human creativity.^14^ One of the most tested approaches has been the creative cognition model.^15^ According to this approach, which is to some extent consistent with the Shared Vulnerability Model, creativity (i.e., conceived as the generation of original and useful ideas) emerges from the application of basic cognitive functions to already existing knowledge structures.^15^ This means that several cognitive processes could underlie creative thinking to some extent.^15^ Specifically, executive functions (EF) have been the most studied processes to date.^16^

Studies carried out among healthy people indicate that a greater performance in EF is related to better creative thinking.^16^ However, some inconsistencies have been found across studies, suggesting that different components of EF may be necessary for different creative abilities.^17^ For instance, regarding inhibition, creativity has been related not only to higher inhibitory control,^16^, ^18^, ^19^, ^20^ but also to worse inhibitory control,^21^, ^22^ as well as to adaptive and flexible inhibitory control.^23^, ^24^ These inconsistent findings may be due to the different cognitive demand of creative tasks. In other words, some creative tasks may require more focused attention and higher inhibition for better performance (e.g., to suppress salient and less original ideas), whereas others may require just the opposite (defocused attention and disinhibition).^18^ Based on this idea, creative people may have greater flexibility in cognitive control,^24^ so they may be able to adjust their attention more easily according to the demands of the task at hand.^18^ The relation between creativity and CF has received little study and both significant^24^, ^25^, ^26^ and non‐significant associations^16^ have been reported. According to Pan and Yu,^25^ this inconsistency may be due to the fact that different creativity dimensions are measured in each study. These authors^25^ found that CF was related to the fluency and flexibility dimensions of creativity, but not to originality. Benedek *et al*.,^16^ who only measured the originality dimension of creativity, did not find any relation between CF and creativity.

WM is another element of EF necessary for creative thinking, as it enables the maintenance of innovative information in an elevated state of activity and the distinction between relevant or irrelevant information for the task.^27^ Although not many studies have analyzed this relation, these have found a positive association between creativity and WM among healthy people.^16^, ^27^, ^28^

Taking into account that, on the one hand, all this research suggests that various dimensions of EF are important processes involved in creative performance and, on the other hand, people with schizophrenia have impaired EF,^29^ schizophrenia may be expected to be related to worse creative performance partially due to impairment in these cognitive functions. As far as the authors are aware, only two studies have analyzed the relation between creativity and EF in schizophrenia,^4^, ^5^ and they found worse performance in these domains in schizophrenia compared to HC. Only one of these studies^4^ explored the mediatory role of EF in creativity, and it found that they played a mediatory role in creative performance in this disease.

Despite not being included in the Shared Vulnerability Model, theory of mind (ToM) is another function that is altered in schizophrenia.^30^, ^31^ *ToM* has been defined as the ability to impute mental states to others.^32^ Although a paucity of studies exists, so far evidence has suggested that ToM and creativity, concretely divergent thinking, are significantly related to each other in healthy people.^33^, ^34^, ^35^ A divergent thinking task requires actively searching one's knowledge, which implies knowing what one knows.^34^ In contrast, a ToM task requires knowing what others know. According to Suddendorf and Fletcher‐Flinn,^35^ these two skills (i.e., knowing what one knows and knowing what others know) are closely related to each other, as both entail meta‐representational thinking. More specifically, Suddendorf and Fletcher‐Flinn found that it was the ToM capacity that predicted an improvement in divergent thinking. It is thought that the meta‐representational skills involved in ToM may not only be important for understanding other people's minds, but also for accessing and scanning one's own mind.^35^ Thereby, when an individual is able to meta‐represent, then that individual is able to see information from multiple perspectives and thus to simultaneously entertain different representations of the same object (and produce different uses for it) and to consider various alternative solutions for a problem. Therefore, capacities such as divergent thinking, which apparently depend upon accessing one's mind, may improve with the acquisition of ToM.^35^ Additional evidence of this relation comes from neural substrates of ToM in schizophrenia^36^, ^37^, ^38^ and creativity,^39^ including the inferior frontal gyrus, medial temporal lobe, anterior cingulate cortex, inferior parietal lobe, or precuneus. Moreover, the default mode network seems to be involved in both creative thinking^40^ and ToM.^41^ However, as far as the authors are aware, this relation has not been explored among patients with schizophrenia.

All this evidence suggests that the ability to perform creative tasks could be altered among people with schizophrenia, at least partially, due to impairment in EF and ToM. Therefore, the first objective of this study was to analyze differences in various dimensions of creativity in patients with schizophrenia and HC. The second aim was to assess whether EF and ToM mediate the relation between schizophrenia and different creative abilities. First, it was hypothesized that patients with schizophrenia would obtain lower scores in creativity compared to HC. Second, it was hypothesized that the poorer creative performance among patients with schizophrenia would be at least partially due to deficits in EF and ToM.

## Methods

### Participants

The sample consisted of 45 patients diagnosed with schizophrenia (35 males, mean age 41.58 years [SD = 8.67 years], and mean education 10.56 years [SD = 2.86 years]) who were recruited from the Psychiatric Hospital of Álava and the Mental Health Network in Álava, Spain, together with 45 HC (15 males, mean age 38.91 years [SD = 14.67 years], and mean education 14.67 years [SD = 3.58 years]). All patients met the diagnostic criteria for schizophrenia according to the Structured Clinical Interview for DSM‐IV‐TR.^42^ Mean illness duration was 18.68 years (SD = 8.62 years) with a mean medication dosage (chlorpromazine‐equivalent doses) of 421.99 mg/day (SD = 255.58 mg/day). Medication was changed to chlorpromazine by using the defined daily dose method.^43^, ^44^ Regarding clinical symptoms (measured with the Positive and Negative Syndrome Scale [PANSS]^45^), patients showed a mean score of 12.50 (SD = 4.35) on the Positive subscale, 21.45 (SD = 6.50) on the Negative subscale, 32.18 (SD = 8.56) on the General Psychopathology subscale, and 66.13 (SD = 16.04) on the total score.

Exclusion criteria consisted of: (i) clinical instability (total score on PANSS‐Positive >19); (ii) cognitive impairment secondary to another disease; (iii) main diagnosis of substance use disorder or presenting active drug consumption at the time of the study; (iv) important modifications to the antipsychotic drug treatment in the previous 3 months; and (v) diagnosis of an active major affective disorder. The study protocol was approved by the Clinical Research Ethics Committees of the Autonomous Region of the Basque Country (CEIC‐E) in Spain (PI2017044). The trial was registered in http://clinicaltrials.gov (NCT03509597). Healthy controls were additionally recruited for this specific study. All participants took part in the study voluntarily and provided written informed consent to participate in the study. Participants did not receive any monetary reward for taking part in the study.

### Measures

#### Executive functions

Two components of EF were measured: WM and CF. CF was assessed with the Stroop Test.^46^ A composite score obtained from Stroop Word‐Color and Stroop Interference values (Cronbach's α = 0.90) was used. WM was measured with the Backward Digit Span subtest from the Wechsler Adult Intelligence Scale – III.^47^

#### Social cognition

ToM was measured by means of the Spanish version of the Happé Test ‘Strange Stories Task,’^48^ developed by Pousa.^49^ The test is composed of stories concerning double bluff, mistakes, persuasion, and white lies. The test involved reading and then answering a question from each story. The questions required making an inference about the character's thoughts and intentions. Each response was scored within a range of 0–2. Explicit answers had a 2‐point score, implicit answers a 1‐point score, and no response or non‐related responses scored 0 points. Four stories were used, and a total score from the four stories was obtained ranging from 0 to 8, with higher scores indicating better performance.

#### Creativity

Two subtests from the Torrance Test of Creative Thinking were used for assessing creativity.^50^ From the Figural Form of the test, the Picture Completion subtest was employed. In this activity, participants were asked to complete 10 unfinished figures, generating as many ideas as possible. Several dimensions were measured: originality, fluency, elaboration, resistance to premature closure, abstractness of titles, and creative strengths. These dimensions were calculated using the Torrance Test of Creative Thinking scoring manual.^50^ Originality was defined as the ability to produce unusual or statistically infrequent responses. Responses were classified as original (1 point) or unoriginal (0 points) according to a list that had been developed for each item on the basis of normative data.^50^ Fluency was measured by the number of relevant responses produced, that is, the number of figures completed. Each completed figure was assigned 1 point. Elaboration was defined as the number of things added to a figure that were considered to be details. Each detail was assigned 1 point. Resistance to premature closure was assessed based on the ability to quickly resist closing the incomplete figures. Scores ranged from 0 (quick closure and no resistance to closure) to 2 (incidental or no closure, most resistance to closure) for each picture. Abstractness of titles measured the degree to which a title moved beyond concrete labeling. Each title was scored on a four‐point scale (0–4). The lowest score was awarded when the title only identified the picture using an obvious class or generic title, while the highest score was given when the title was abstract and captured the essence of the information involved. Creative strengths were assessed by 11 criterion‐referenced measures: emotional expressiveness, storytelling articulateness, movement or action, expressiveness of titles, synthesis of incomplete figures, unusual visualization, internal visualization, humor, richness of imagery, colorfulness of imagery, and fantasy. Each creative strength portrayed was awarded 1 point. Additionally, the flexibility dimension was measured according to the criteria from the Spanish adaptation of the Torrance Test of Creative Thinking.^51^ Flexibility was the production of different ideational categories. Each picture was classified according to the corresponding category, using the list of categories from the Spanish adaptation of the Torrance Test of Creative Thinking.^51^ One point was given for each different category used. The scores obtained in each dimension of each picture were summed to obtain a total score for each dimension. Moreover, a total figural creativity score was calculated by using the sum of the scores for originality, elaboration, fluency, resistance to premature closure, abstractness of titles, and flexibility.

The Unusual Uses subtest was administered from the Verbal Form of the test. In this test, participants were asked to write all of the unusual uses for Cardboard Boxes that they could think of. Three dimensions were measured: originality, fluency, and flexibility. Originality was scored according to the items listed in the manual,^50^ awarding 1 point for original or uncommon responses, and 0 points for unoriginal responses. Fluency was the total number of unusual uses produced, where 1 point was assigned to each unusual use. Flexibility was the number of different categories used, awarding 1 point to each category. A total verbal creativity score was calculated with these three dimensions. Finally, according to the Torrance Test of Creative Thinking scoring system,^50^ a total creativity score was obtained (Cronbach's α = 0.90). Participants were given 4 min to complete each creative activity.

An expert neuropsychologist corrected all the tests. In addition, a second neuropsychologist corrected the creativity tasks for a subsample of 32 participants (inter‐rater reliability ranged from 0.89 to 0.99).

#### Clinical symptoms

Psychopathology was assessed by using the PANSS Positive, Negative, and General Psychopathology subscales.^45^

### Data analyses

Statistical analyses were carried out with spss Version 24.0 (IBM, Armonk, NY, USA). Data were tested for normality using the Shapiro–Wilk test. The χ^2^ test was used to analyze differences between the two groups according to sex. Spearman's Rho correlations were performed between neuropsychological variables and illness duration. Differences between groups on sociodemographic variables were assessed by a two‐tailed independent *t*‐test. The differences between the groups in EF, ToM, and creativity variables were performed by an analysis of covariance in order to control for possible interaction of several covariates. Significance level was set at 0.05. Effect sizes were obtained through partial eta squared ($\eta_{p}^{2}$). This was interpreted as small (0.01), medium (0.06), and large (0.14).^52^

The mediation hypothesis was tested by using path analysis with lisrel 9.2.^53^ The robust maximum likelihood (RML) method was employed, which requires an estimate of the asymptotic covariance matrix of the variances and covariates of the sample and includes the scaled χ^2^ Satorra–Bentler index. Missing values were imputed using the expectation maximization algorithm. The goodness of fit of the model was evaluated by the root‐mean‐square error of approximation (RMSEA), comparative fit index (CFI), non‐normed fit index (NNFI), and standard residual mean square root (SRMR). According to Hu and Bentler, CFI values higher than 0.90, RMSEA values smaller than 0.06, and SRMR values smaller than 0.08 reflect a good fit.^54^

Participants from the present study were assured raw data would remain confidential and would not be shared. Therefore, data of the study are not available in the public domain.

## Results

Regarding sociodemographic variables, statistically significant differences between groups in sex distribution (χ^2^ = 1.05, *P* < 0.001) and years of education (*t* = −6.01, *P* < 0.001), but not in age (*t* = 1.05, *P* = 0.297) were found. In order to control for their possible influence, these three variables were introduced as covariates in the subsequent analyses. Additionally, correlation analyses were performed between illness duration and CF, WM, ToM, and creativity. Significant correlations were not found between illness duration and CF (Spearman's Rho = 0.067, *P* = 0.680), WM (Spearman's Rho = −0.088, *P* = 0.590), ToM (Spearman's Rho = 0.200, *P* = 0.215), figural creativity (Spearman's Rho = 0.179, *P* = 0.269), verbal creativity (Spearman's Rho = 0.257, *P* = 0.109), and total creativity (Spearman's Rho = 0.133, *P* = 0.414). Therefore, illness duration was excluded from further analyses.

### Differences in EF, ToM, and creativity between groups

An analysis of covariance was performed in order to assess the differences between the two groups in EF, ToM, and creativity, controlling for the possible effect of sex, age, and years of education. Differences between the two groups in terms of EF, ToM, and creativity can be found in Table 1.

**Table 1:** Mean differences between groups in EF, ToM, and creativity (multivariate analysis of covariance)

	Schizophrenia (*n* = 45) M (SE)	HC (*n* = 45) M (SE)	*F*	Effect size ($\eta_{p}^{2}$)
EF				
WM	5.61 (0.34)	7.68 (0.31)	18.86**	0.183
CF	−0.35 (0.15)	0.30 (0.14)	9.37*	0.100
ToM	3.90 (0.38)	6.53 (0.35)	23.82**	0.221
Figural creativity				
Figural originality	2.77 (0.39)	3.19 (0.36)	0.57	0.007
Figural fluency	6.37 (0.43)	6.80 (0.39)	0.51	0.006
Figural elaboration	19.16 (1.94)	22.63 (1.77)	1.59	0.019
Figural flexibility	5.22 (0.33)	5.74 (0.30)	1.26	0.015
Figural resistance to closure	10.03 (0.74)	11.30 (0.67)	1.47	0.017
Figural abstractness of titles	5.41 (0.79)	8.97 (0.72)	10.09*	0.107
Figural strengths	3.63 (0.55)	5.57 (0.50)	6.19*	0.069
Figural creativity	48.96 (3.25)	58.63 (2.98)	4.38*	0.050
Verbal creativity				
Verbal originality	4.85 (0.77)	7.27 (0.70)	4.92*	0.055
Verbal fluency	8.13 (0.89)	12.76 (0.81)	13.47**	0.138
Verbal flexibility	5.37 (0.53)	8.13 (0.48)	13.43**	0.138
Verbal creativity	18.36 (2.06)	28.16 (1.89)	11.49**	0.120
Total creativity	3.42 (0.60)	5.80 (0.54)	7.92*	0.086

* *P* ≤ 0.05.

** *P* ≤ 0.001.

Age, years of education, and sex were entered as covariables. Figural creativity and verbal creativity variables are the sum of each figural and verbal dimension, respectively. Total creativity is the average of all creative scores plus creative strengths.

CF, cognitive flexibility; EF, executive functions; HC, healthy controls; M, estimated marginal means; SE, standard error; ToM, theory of mind; WM, working memory.

Regarding figural creativity measures, HC obtained higher scores in all the variables compared to patients with schizophrenia. However, statistically significant differences were only found in the: figural abstractness of titles (*P* = 0.002), showing a medium effect size ($\eta_{p}^{2}$ = 0.107); figural strengths (*P* = 0.015), with a medium effect size ($\eta_{p}^{2}\;$= 0.069); and in the total figural creativity score (*P* = 0.039), with a small effect size ($\eta_{p}^{2}$ = 0.050). Regarding verbal creativity scores, HC obtained higher scores compared to individuals with schizophrenia. These differences were statistically significant for all variables of verbal creativity. Effect sizes were medium for verbal fluency (*P* = 0.000, $\eta_{p}^{2}\;$= 0.138), verbal flexibility (*P* = 0.001,$\;\eta_{p}^{2}$ = 0.138), and total verbal creativity (*P* = 0.001,$\;\eta_{p}^{2}$ = 0.120), and small for verbal originality (*P* = 0.029,${\;\eta }_{p}^{2}$ = 0.055). Significant differences were found in the total creativity score (*P* = 0.006), with higher scores among HC again. The effect size was medium ($\eta_{p}^{2}$ = 0.086).

Turning to EF, statistically significant differences were found between groups in CF (*P* = 0.003), with HC obtaining higher scores. Effect size was medium ($\eta_{p}^{2}$ = 0.100). Regarding WM, HC obtained significantly higher scores than patients with schizophrenia (*P* = 0.000) and effect size was high ($\eta_{p}^{2}$ = 0.183). With regard to ToM, HC also showed significant higher scores in comparison to patients with schizophrenia (*P* = 0.000); effect size was high ($\eta_{p}^{2}\;$ = 0.221).

### Structural equation model explaining group differences in verbal and figural creativity

Path analysis was used in order to assess whether group differences in creativity were mediated by CF, WM, and ToM. Additionally, as significant results had been obtained in the preliminary analysis of covariance, age and years of education were included in the model in order to control for their possible effect.

Results showed that, on the one hand, schizophrenia was significantly and negatively associated with CF, WM, and ToM. On the other hand, CF was significantly and positively related to figural creativity, ToM was positively and significantly related to both figural and verbal creativity, and WM was positively and significantly associated with both verbal and figural creativity. The fit of the model was very good, χ^2^ (9, *N* = 90) = 13.11, RMSEA = 0.072 (90% confidence interval: 0.00–0.15), CFI = 0.99, NNFI = 0.97, and SRMR = 0.046. In order to test the significance of mediations, Sobel's test was performed. This was statistically significant for several mediations. Specifically, ToM played a mediatory role in the relation between group (being a patient with schizophrenia or an HC), and both figural (*Z* = 2.075, *P* = 0.037) and verbal creativity (*Z* = 2.570, *P* = 0.010); WM mediated the relation between group and figural creativity (*Z* = 2.034, *P* = 0.041) and had a tendency to significance in verbal creativity (*Z* = 1.930, *P* = 0.053); and finally, CF mediated the relation between group and figural creativity (*Z* = 2.454, *P* = 0.014).

**Figure 1:**
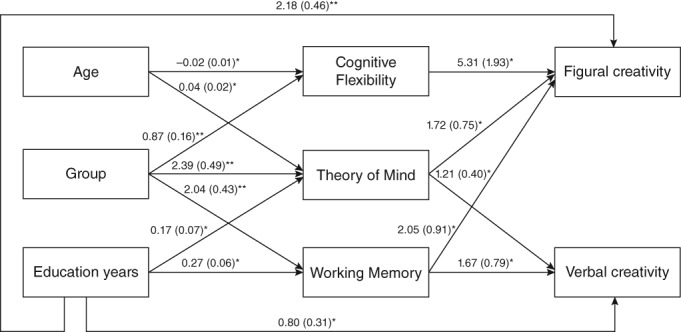
Model of the mediation of cognitive flexibility, working memory, and theory of mind between group and creativity, between education years and creativity, and between age and creativity. Given values are non‐standardized coefficients with standard errors in parentheses. *P ≤ 0.05. **P ≤ 0.001.

As can be observed in Figure 1, not having a diagnosis of schizophrenia was associated with better performance in CF, WM, and ToM, and this better performance was associated with higher scores in verbal and figural creativity. In both figural and verbal creativity, there were statistically significant full mediations.

## Discussion

The objective of this study was to analyze differences in various dimensions of creativity between patients with schizophrenia and HC, as well as to assess the possible mediating role played by EF (CF and WM) and ToM in this relation. The main hypothesis of the study was partially confirmed; that is, patients with schizophrenia obtained significantly lower scores in all creativity variables, except for some of the figural creative variables. The fact that statistically significant differences were found in all the verbal creative variables, but not in all the figural creative variables, is in line with a recent meta‐analysis.^9^ This could be due to the language dysfunction that is present in schizophrenia, such as formal thought disorder or alogia.^55^, ^56^ This language dysfunction has been shown to have a negative impact on tasks involving verbal fluency.^56^ As the verbal creativity tasks imply a verbal fluency component, performing these kinds of activities could be particularly difficult for people with schizophrenia. Other studies have also found lower scores in schizophrenia compared to HC in verbal creative tasks^4^, ^57^, ^58^, ^59^ as well as no statistically significant differences in some figural creative variables.^4^, ^59^ In addition, as expected, patients with schizophrenia obtained lower scores in CF, WM, and ToM compared to HC. This finding was also consistent with results obtained in previous research.^29^, ^30^

The second main hypothesis was that, according to the Shared Vulnerability Model,^10^ the initial differences between HC and patients with schizophrenia in creative performance would be, at least partially, due to the impairment in EF observed in schizophrenia. Given the previous evidence of the relation between ToM and creativity among healthy people,^33^, ^34^, ^35^ along with the underlying shared brain structures^36^, ^37^, ^39^ and the impairment shown in ToM in schizophrenia,^30^, ^31^ we decided to analyze whether ToM played a mediatory role in the relation between schizophrenia and creativity. This hypothesis was also confirmed. Differences in creative performance between patients with schizophrenia and HC were mediated by their performance in EF (both CF and WM) and ToM. With regard to WM, this cognitive domain mediated the relation between group and figural creativity and had a tendency towards significance in verbal creativity. With respect to CF, it was found that it mediated the relation between group and figural creativity. The comparison of these results with previous studies is difficult, as few studies have analyzed the relation between EF and creativity in people with schizophrenia.^4^, ^5^ In particular, Jaracz *et al*.^5^ found that lower scores in the creativity of patients with schizophrenia compared to HC correlated with lower scores in EF. Partly in line with our results, Abraham *et al*. found that various components of EF mediated differences in creativity between people with schizophrenia and HC.^4^ The mediatory effect of EF (CF and WM) observed in our study is consistent with previous findings, and supports the hypothesis of the Shared Vulnerability Model proposed by Carson.^10^

Studies performed among healthy people have also found an association between creativity and EF.^16^, ^18^, ^20^, ^24^, ^27^ Benedek *et al*. analyzed the association between inhibition, CF, WM updating, and various verbal creativity tasks and found that it was the WM variable that most strongly predicted verbal creativity. Similarly, de Dreu *et al*. found a relation between WM and creativity.^27^ Benedek *et al*. showed that total creativity (measured with several verbal and figural tasks) correlated positively and significantly with inhibition.^20^ Likewise, Edl *et al*. found an association between cognitive control and both verbal and figural creativity.^18^ In addition, Zabelina and Robinson identified a relation between CF and verbal and figural creativity.^24^ All these studies suggest that multiple components of EF, such as WM, inhibition, and CF, are involved in creativity.

ToM mediated the relation between group and both figural and verbal creativity. It is worth noting that ToM played a mediatory role even after controlling for the effect of CF, WM, age, and years of education. As far as the authors are aware, very few studies have explored the relation between ToM and creativity and none of them has analyzed this relation in schizophrenia. Three studies explored this relation among healthy children^33^, ^34^, ^35^ and found a positive association. Specifically, Suddendorf and Fletcher‐Flinn found a positive correlation between ToM and verbal creativity.^34^, ^35^ Moreover, Sigirtmac identified a positive relation between ToM and figural creativity.^33^ These results suggest that participants’ meta‐representational skills that are involved in ToM are associated with figural and verbal creative performance. These meta‐representational skills may permit an individual to see information from multiple perspectives and consider different alternative solutions to a problem, namely, improve divergent thinking. Additionally, these findings seem to be reinforced by the fact that the default mode network is involved in both creative thinking^40^ and ToM.^41^ The involvement of ToM in this relation between schizophrenia and creativity is very interesting and promises to be a new field of research in which ToM, or even other dimensions of social cognition, could be considered to be possible protective factors in the Shared Vulnerability Model.^10^ Specifically, these findings suggest that increased accessibility to associational material and unusual thoughts may not only require executive control to increase creativity, but also the ability to understand that something can be represented in different ways, that is, the ability to meta‐represent. This capacity may enable the individual to sense all of the unusual and bizarre ideas that are mentally processed, and allow these thoughts to be taken advantage of (without being confused by them), which can protect against psychopathology. In contrast, difficulty in understanding that things are represented and that other people have different thoughts (that is, to meta‐represent) may impede the control and manipulation of unusual thoughts, and therefore, cause people to be overwhelmed or confused by them, and increase the risk of developing a psychopathology.

This study has several limitations that should be considered. First, patients with schizophrenia and HC did not match in terms of sex and years of education. Therefore, their possible effect was controlled for by entering them as covariates. Second, all patients with schizophrenia were receiving antipsychotic treatment, so the possible effect of the medication on the performance of the different tasks could not be controlled for. Third, it was not possible to include all the variables proposed in the Shared Vulnerability Model, such as IQ.

Bearing these limitations in mind, we believe that the results obtained are quite important, as they support and extend the Shared Vulnerability Model.^10^ Future studies could explore the relation between creativity and ToM among the unaffected relatives of patients with schizophrenia, or among high schizotypal people. Furthermore, it would be interesting to study the possible role of other social cognitive functions in this relation. In addition, these findings raise some new, interesting questions for future studies, such as whether an improvement in cognitive functions would lead to improved creative thinking. Specifically, whether the implementation of interventions focused on ToM, in addition to other cognitive functions, such as EF, may enhance creativity. Moreover, considering the role that creativity plays in problem solving in daily life,^60^ could the enhancement of creative thinking improve the daily functioning of people with schizophrenia? All these issues should be addressed by future research studies.

## Disclosure statement

The authors declare no conflict of interest.

## Author contributions

Authors N.O., N.I.B., J.P., P.S., N.I.Y., and M.T.E. designed the study and wrote the protocol. Authors A.S., S.L.G., P.S., and N.I.Y. performed the clinical and neuropsychological evaluations. A.S. and J.P. managed the literature searches and undertook the statistical analysis. A.S. and J.P. wrote the first draft of the manuscript. All authors contributed to the writing and revision of the manuscript. All authors have approved the final manuscript.
